# Editorial: Roles of macrophages and monocytes in resistance to immunotherapy in cancers

**DOI:** 10.3389/fimmu.2026.1784612

**Published:** 2026-01-19

**Authors:** Guillaume Mestrallet, Susanna Mandruzzato

**Affiliations:** 1 Division of Hematology and Oncology, Hess Center for Science & Medicine, Tisch Cancer Institute, Icahn School of Medicine at Mount Sinai, New York, NY, United States; 2 Department of Surgery, Oncology and Gastroenterology, University of Padova, Padova, Italy; 3 Immunology and Molecular Oncology Unit, Veneto Institute of Oncology (IOV)-Istituto di Ricovero e Cura a Carattere Scientifico (IRCCS), Padua, Italy

**Keywords:** cancer, immunotherapy, macrophages, myeloid cells, resistance

Macrophages and monocytes are key architects of the tumor immune microenvironment and central mediators of resistance to immunotherapy. Tumors exploit the remarkable plasticity of these myeloid cells to suppress T-cell responses, remodel metabolic niches, and promote progression. This Research Topic brings together one original research article and three review articles that collectively illuminate how myeloid cells drive immune escape and how they can be therapeutically reprogrammed to overcome resistance.

The original research article by Luo et al. investigates hepatocellular carcinoma (HCC) and uncovers a metabolic mechanism by which tumor cells reshape macrophage behavior. The authors identify fatty acid-binding protein 5 (FABP5) as a cargo selectively packaged into exosomes released by HCC cells. These exosomes deliver FABP5 to macrophages, inducing lipid accumulation via activation of PPARγ signaling and suppression of fatty acid oxidation. This metabolic rewiring skews macrophages toward an immunosuppressive phenotype. *In vivo*, loss of FABP5 leads to reduced tumor growth, diminished accumulation of immunosuppressive TAMs, and enhanced antitumor immunity. This study highlights the capacity of tumor-derived extracellular vesicles to instruct macrophage identity and identifies FABP5 as a targetable driver of myeloid-mediated immune resistance.

The first review (Wang et al.) provides a comprehensive overview of TAM biology, revisiting the classical M1/M2 polarization framework while emphasizing the diverse and dynamic activation states revealed by modern single-cell profiling approaches. The review highlights that TAMs largely derive from recruited monocytes and acquire diverse pro-tumoral functions, including suppression of T-cell activity, promotion of invasion, and enhancement of metastatic dissemination. In addition, it summarizes current therapeutic strategies aimed at depleting, reprogramming, or enhancing the phagocytic activity of TAMs, underscoring their central role in shaping responses to immunotherapy.

A second review (Gong et al.) focuses on pancreatic ductal adenocarcinoma (PDAC), one of the most immunotherapy-resistant cancers. The authors detail how suppressive myeloid populations dominate PDAC tumors, and how these programs differ between primary tumors, pre-metastatic liver niches, and established metastases. This stage-specific myeloid biology provides important insight into the limited efficacy of immunotherapy in PDAC and the frequent mismatch between preclinical and clinical responses. The review highlights emerging therapeutic strategies, including CD40 agonists and CSF-1R inhibitors that aim to reprogram myeloid cells and improve immune responsiveness of this notoriously resistant tumor type. The final review (Shang et al.) examines the CSF-1/CSF-1R pathway, a central regulator of macrophage differentiation, with particular emphasis on its relevance to radiotherapy. Radiotherapy often induces an immunosuppressive macrophage response that limits antitumor immunity and restricts systemic, abscopal effects. Inhibiting CSF-1R can reduce suppressive TAM accumulation, enhance radiotherapy efficacy, and mitigate radiation-induced fibrosis. This review synthesizes recent advances in CSF-1R-directed therapeutics and highlights its potential as a combination partner to improve responses to both radiotherapy and immunotherapy.

Together, the contributions in this Research Topic converge on several key principles: tumors actively shape macrophage metabolism and identity; myeloid-mediated suppression varies across tumor types and metastatic stages; and targeted modulation of macrophage pathways can enhance the efficacy of existing treatments. By integrating mechanistic insights with emerging therapeutic approaches, these articles collectively underscore the promise of macrophage- and monocyte-centered strategies to overcome resistance to immunotherapy and improve patient outcomes.

